# Psychological Distress in Low-Income and Economically Marginalized Populations in India: Protective and Risk Factors

**DOI:** 10.3390/bs14020092

**Published:** 2024-01-26

**Authors:** Dipti Singh, Shagufta Nasir, Juhi Sharma, Lydia Giménez-Llort, Mohammad Ghazi Shahnawaz

**Affiliations:** 1 Department of Psychology, Jamia Millia Islamia, New Delhi 110025, India; diptisingh2712@gmail.com; 2 Department of Psychiatry and Forensic Medicine, School of Medicine, Universitat Autònoma de Barcelona, 08193 Barcelona, Spain; shagufta.shagufta@autonoma.cat (S.N.); lidia.gimenez@uab.cat (L.G.-L.); 3 Light Up-Emotions Matter Foundation, New Delhi 110096, India; juhi.sharma89@gmail.com; 4 Institut de Neurociències, Universitat Autònoma de Barcelona, 08193 Barcelona, Spain

**Keywords:** mental health, low-income and economic marginalization (LIEM), socioeconomic status, urban slum, gender, caste, intersectionality

## Abstract

Studies at the juncture of development economics and public health take on considerable responsibility in addressing inequality and related mental health distress. Mental healthcare in economically marginalized populations requires depicting the linkages between socioeconomic status and psychological distress. In the present work, a sequential mixed-methods design was used to study 190 people in such communities in India. Gender-dependent psychological distress was found according to the Kessler Psychological Distress Scale (K-10) with moderate distress in women (M = 26.30, SD = 9.15) and mild distress in men (M = 21.04, SD = 8.35). Regression analysis indicated that gender significantly predicted psychological distress, followed by age, marital status, and the level of education of the head of the family. The Interpretative Phenomenological Analysis of semi-structured interviews of the six women who scored the highest on the distress scale unveiled three master themes: (1) manifestation of psychological distress, (2) contextual challenges, and (3) sources of strength and resilience. Overall, participants reported a lack of resources, community violence, gender discrimination, and widespread substance use as major contributors to the ongoing distress. These findings can pave the way for future studies to expand beyond independent economic indicators and curate clinical interventions for culturally competent mental healthcare.

## 1. Introduction

Psychology as a discipline has been at the receiving end of considerable backlash because of research largely affected by sociodemographic and socioeconomic biases and the assumption that “one model fits all”. Thus, studies based on Western populations—educated, industrial, rich, and democratic people (the so-called WEIRD populations) [1]—may suffer from a restrained cultural relevance of their research findings [2]. Therefore, to make psychology more inclusive, the APA Guidelines for Psychological Practice for People with Low-Income and Economic Marginalization (LIEM) were introduced in 2019 [3]. LIEM refers to “an umbrella term encompassing limited financial resources and marginalization related to class”. Moreover, mental health professionals have been criticized for being underrepresented in addressing the growing global social inequalities [4]. Therefore, it is imperative to document the experiences of communities that have been marginalized and deserve the requisite clinical attention. This calls for increased access to quality mental healthcare and culturally appropriate training through a socially inclusive lens. Moreover, without taking into account the myriad of factors, mental healthcare ceases to work effectively with a diverse set of population [5].

Research across various disciplines has shown the implications of economic marginalization on health and well-being [6,7]. There is evidence that low-income spaces are characterized by rampant poverty, poor infrastructure, community violence, and limited career opportunities that further exacerbate the vulnerability to developing mental health issues [8]. In India (the site of the present research), the severity of the mental health burden is alarming and calls for an urgent need for action [9]. According to a 2017 report, one in every seven people in India is affected by mental illness, ranging from mild to severe symptoms [10]. It is imperative to observe that human beings interact in multiple-locked, interconnected systems (age, gender, social class, race/ethnicity, caste, migration, etc.) and power hierarchies. The complexities of these social identities and power structures generally establish the experiences of oppression and privileges in society [11]. Intersectional inequalities are evident in healthcare utilities and are estimated to have a significant effect on mental health outcomes [12]. Moreover, unemployment in India has risen considerably due to the COVID-19 pandemic, as around 1.5 million Indians lost their jobs during this period [13]. Consequently, systematic inequalities have only widened the disparities, with limitations in access to quality mental healthcare. Thus, it is important to study socioeconomic status (SES) across age, gender, caste, and class in a culturally diverse country like India. The present study is a modest attempt in this direction, as it aims to explore the linkages between socioeconomic status (SES) and psychological distress among LIEM populations in India.

Psychological distress is considered an important indicator of any pathological condition. It refers to the “general concept of maladaptive psychological functioning in the face of stressful life events” [14]. Psychological distress is a transient and modifiable non-specific factor of stress, anxiety, and depression that can lead to impaired mental health in individuals [15]. It can be understood as a form of emotional suffering with somatic and psychological symptoms bearing an effect on social functioning [16]. Psychological distress is more evident in highly vulnerable socioeconomic populations and those individuals facing social inequalities [17] The APA Resolution on Poverty and Socioeconomic Status (2000) posits that the mental health risk is higher for racial and ethnic minorities, older adults, refugees and immigrants, persons with disabilities, individuals identifying as LGBTQIA, single mothers, foster children, and people with mental illness [4,18].

Low socioeconomic status (SES) is highly associated with frequent mental health issues as compared to high SES [19]. There is evidence that poverty and income inequality are not only associated with psychological distress but also with other mental illnesses, including depression [20], suicide [21], and schizophrenia [22]. Interestingly, “the study of poverty and mental health in low and middle-income countries is relatively young” [23], and to the best of our knowledge, systematic measurement of psychological distress in economically marginalized communities has been a relatively less-explored area in the Indian context as well [24].

Socioeconomic status (SES) is an elusive term in the psychological literature, as both theoretical and methodological issues are complicated [25]. Socioeconomic status (SES) is a critical aspect of research in other social sciences as well, particularly in the fields of economics and sociology [26]. While the terms social position, social class, and SES are often used synonymously, there are nuanced distinctions between them. This lack of conceptual clarity and limited focus given to SES within psychology has restricted the scope of the application of research findings [3]. In many low- and middle-income countries (LMICs), including India, there has been a focus on objective measures of SES, like poverty [27]. However, other studies have used multiple SES indicators, like household and education [28] and property ownership [29]. The APA Taskforce on Socioeconomic Status (2007) suggested a “triumvirate” measure of income, education, and employment as the most appropriate measure of SES [4]. The income indicator is related to the average total household income of the family; the education index is reflected in the highest level of education attained by the head of the family; and the employment index is the current status of work employment and associated job roles for the family members. Each of these three measures is multidimensional in itself, and thus choosing the most appropriate indicator should account for the outcome of interest. 

Considering India’s social stratification and cultural diversity, it is imperative to focus on other sociodemographic factors, and therefore any analytical framework needs to incorporate caste as well for a better understanding [30] Caste hierarchies and stratification influence social roles and occupations [31], along with deeply entrenched discrimination and social exclusion [32]. Social discrimination, such as caste discrimination, was found to be related to poor mental health as well [33,34]. While caste continues to be a pervasive issue, the empirical data on caste inequalities and mental health demonstrate little representation in India [24,32]. Therefore, there is a need for psychological research and practice to be informed by the perspective of the marginalized communities that are under-represented, under-served, and most importantly, severely distressed.

Thus, the present research aims to explore the relationship between various indices of SES and psychological distress in LIEM communities situated in the National Capital Region of Delhi, India (NCR). This study used a sequential mixed-methods design. Here, a mixed-method approach will not only facilitate our understanding of the nature of distress but also help us delve deep into the precedents and the lived experiences of the people. 

## 2. Materials and Methods

### 2.1. Sample

Data were collected from 190 respondents aged above 18 years from LIEM communities in New Delhi, using purposive sampling. Participants were informed about the purpose of the study, and informed consent was obtained before conducting the data collection. The demographic details of the sample are illustrated in the Section 3. 

For the qualitative investigation, the 10 participants who scored the highest on the Psychological Distress Scale (Kessler-10) [35] were selected, of whom 6 agreed to the interviews. All six of the participants selected for interviews were women and belonged to ST/SC backgrounds, the details of which are presented in the Section 3. 

### 2.2. Measures

Socioeconomic status: To measure SES, the Modified Kuppuswamy SES scale 2021 was used [36]. It was created by Kuppuswamy in 1976 and modified in 2021 to accommodate the socioeconomic changes in the country over the years. It consists of three indices of SES: education, employment, and income. Moreover, a total score of SES can be obtained by combining all three indices.

Caste: In the sociodemographic form, the subcategories for caste were taken from Pareek’s Revised Scale—2019 [37]. It measures caste in the categories of general caste, agriculture caste, artisan caste, lower caste, schedule caste, and schedule tribe. 

Psychological distress: Kessler Psychological Distress Scale (K-10) was used to measure psychological distress [35]. It consists of ten items with a 5-point scale, ranging from “None of the Time” to “All of the Time”. The items on the scale included, “In the past 4 weeks, how often did you feel depressed?” and “In the past 4 weeks, how often did you feel nervous?” The Cronbach Alpha value for K-10 was found to be 0.90 on the current sample. 

### 2.3. Procedure

Before conducting the main study, K-10 was pilot-tested on 30 respondents from one of the LIEM communities located in the NCR, India. The Cronbach alpha value was found to be 0.83. An a priori power analysis using G*Power version 3.1.9.4 software (Faul, 2019) was used to estimate the sample size to detect a medium effect, f^2^ = 0.15, with 80% power while using linear multiple regression (fixed model and R2 deviation from zero) at an alpha level of 0.05. The estimated sample size was 146, and therefore the actual sample size of the study (N = 190) was adequate to test the hypotheses of the study. Before the initiation of the study, an informed consent form was signed, followed by the demographic form comprising information on age, gender, religion, caste, marital status, hometown, etc. 

Semi-structured interviews were conducted on six women respondents (as mentioned earlier). Informed consent was signed by the participants prior to the interview. Interviews were conducted face-to-face and were recorded using a simple telephonic recorder. The interviews consisted of ten questions, which took about 25–30 min for each participant. The interviews were transcribed verbatim for the analysis.

### 2.4. Design and Analytical Plan

This study employed a sequential mixed-method design [38]. The quantitative data were analyzed using descriptive statistics, correlation, and multiple linear regression. Qualitative interviews were analyzed using the Interpretative Phenomenological Approach (IPA) by Smith et al. (2009) [39]. The IPA is a qualitative approach, at the core of which are the lived experiences of a particular phenomenon. According to Creswell (2013), “phenomenological study describes the common meaning for several individuals of their lived experiences of a concept or a phenomenon” [40] (p. 76). The IPA was chosen for this study given the sensitivity of the context and the rich narratives. While other qualitative approaches are focused on building themes from the data, the IPA allows the experiences to be at the forefront, with a detailed picture of the phenomena [41]. 

## 3. Results

### 3.1. Descriptive Statistics and Correlation

Table 1 shows the demographic details of the study population. Participants had a mean age (±) SD of 32.8 (±) 12.43 years of age. In all, 48.9% were women and 51.1% were men. A majority of 92.1% of participants were practising Hinduism, 7.4% were Muslims, and 0.05% were from other religions. 

In the present study, Table 1 highlights that 73.7% of the participants belonged to ST/SC and other backward caste (OBC) backgrounds, and around 4.2 and 5.8 belonged to artisanal and agricultural castes, respectively. Only 11.6% of the participants belonged to the general category (higher caste). 

For the qualitative study, the demographics details of the participants are shown in Table 2. A total of 10 individuals who scored highest on the Kessler Psychological Distress Scale (K-10) were selected for the interview. All ten participants who scored highest on the distress scale were women and from the SC/ST caste. Out of which, 4 participants refused to be part of the interview schedule. 

The study variables’ mean, standard deviation, skewness, kurtosis, and Pearson correlation for the study variables are presented in Table 3. The normality of data was obtained as the skewness and kurtosis values were within the prescribed range of −2 to +2. However, the kurtosis value was found to be 15.79 for the family income, this is because most of the respondents scored towards the lower side of the income range as they belonged to the LIEM population. 

In Table 3, the results show a significant and positive correlation between gender and psychological distress (r = 0.29, *p* < 0.01), implying that females experience more psychological distress than men. We can also see a significant and positive correlation between age and psychological distress (r = 0.20, *p* < 0.01). There was a significant and negative correlation between the education of the head of the family and psychological distress (r = −0.16, *p* < 0.05). 

### 3.2. Multiple Regression Analysis

As presented in Table 4, about 19% (F = 7.23, *p* < 0.01) of the variance in psychological distress can be explained with the help of predictors, i.e., gender, age, marital status, occupation of the head of the family, education of the head of the family, and income of the family. The effect size for this was reported to be moderate (f^2^ = 0.24), as per Cohen’s (1988) guidelines. Moreover, as indicated by Table 4, gender (β = 0.34, t = 4.95, *p* < 0.001) emerged as the most important predictor, with females being found to report more psychological distress than males. This was followed by age (β = 0.29, t = 3.79, *p* < 0.001), with increased psychological distress being reported with an increase in age, marital status (β = 0.20, t = 2.67, *p* < 0.01), with unmarried respondents reporting higher distress, and finally education levels (β = −0.14, t = −2.03, *p* < 0.05), with lower levels of education of the head of the family predicting higher psychological distress.

### 3.3. Qualitative Analysis

In this section, the IPA analysis of the transcripts is presented in Table 5. The analysis yielded three master themes and seven sub-themes that provided insight into the lived experiences of participants as they pertain to their psychological distress. 

#### 3.3.1. Master Theme 1: Manifestation of Psychological Distress

This theme explains how symptoms of psychological distress were manifested among the participants in both physical and emotional forms.

##### Physiological Disturbances and Bodily Concerns

The disturbances in sleep patterns and lack of appetite were reported by four out of the six participants. They described waking up in the middle of the night due to a sense of restlessness. All six participants also reported extreme fatigue and exhaustion. 

Mrs. R complained of less than adequate sleep in the last two years. She worried excessively while being preoccupied with stressors at night and was also recommended by doctors to relieve some of her tension and anxiety.

Mrs. R: “I can’t sleep properly at night, everyone says that I think too much at night, I feel extremely restless at night and feel like running away from home.”

Mrs. V gave a detailed explanation of her chronic stomach pain, and while she had consulted several doctors for this, no significant medical reasons were identified, and no sense of relief was observed.

Mrs. V: “When I am stressed or worried, I can’t eat. I always have pain in my stomach, but no medicines have helped.”

##### Emotional and Psychological Disturbances

The participants expressed excessive worry about the future and were fearful of losing their jobs during the COVID-19 pandemic. 

Mrs. T was also constantly worried about rationing the food and if her husband would be able to earn enough money to pay the gas bill.

Mrs. T: “The whole month I worry if we can buy enough ration. COVID-19 brought many problems, and we were constantly worrying if someone can at least give INR 10–20 to us.”

In addition to this, one could also observe persistent feelings of sadness, and most importantly, frequent crying spells. At some point in the interview, all participants either softly teared up with the sudden flash of the traumatic incidents or cried profusely, holding the interviewer’s hand for support. Mrs. S teared up at the mention of her childhood, hoping that her children are not put through the same ordeals. 

Mrs. M cried twice during the interview when remembering her deceased son. She also explained her initial symptoms of depression, where she would not move from her place, barely sleep or eat at all, and would just cry at the sight of her daughter-in-law.

Mrs. M: “I was broken from the inside and I had left everything and would just lie on this sofa the whole day crying. My son would say that mother you have gone crazy, what will we do, we have nobody except you.

#### 3.3.2. Master Theme 2: Contextual Challenges 

This theme describes the experiences and challenges of living in an urban slum, which are marked by daily physical and social difficulties. 

##### Experiences of Living in Abject Poverty

The participants explained their daily hassles and the general environment as being riddled with extreme poverty, low education, and limited resources. All six women gave a detailed explanation of their personal experiences of struggling to make ends meet. All the participants had not received formal education and had to drop out of primary school due to financial constraints. 

Mrs. V mentioned that being unemployed was the biggest worry in her life and that the COVID-19 pandemic exacerbated the challenges for them as a family.

Mrs. V: “We both lost our jobs, our children are living hand to mouth, not getting work during the Lockdown has made things even more difficult.”

Mrs. K also expressed their desire to study and said that they would not have suffered so much had they completed their education.

Mrs. K: “I have not studied at all, neither was I sent to school, nor did I go myself. My parents were extremely poor, and we were struggling as a family of eight. None of the children have gone to school, only my parents earned a livelihood, that too through boot polishing.”

In addition to the limited access to resources, all the women respondents complained of an unsafe environment owing to widespread substance use and alcoholism in the community.

Mrs. S: “The biggest problem here are the alcoholics, as they are ruining the future of the children, everyone has access to substances right from small children to older men.”

##### A Spectrum of Violence and Traumatic Incidents

A common element in all the interviews was acts of violence. These ranged from verbal and physical abuse to caste and gender-based violence. The women reported young boys using abusive language and the older men, including their husbands, engaging in domestic violence due to chronic alcoholism. 

Mrs. R explained how, before the death of her husband, there were regular verbal and physical incidents between the couple. Mrs. M also narrated the whole story of how her young son was killed in caste-based violence. Despite overt resistance by the two families, the boy and girl had run away from home and gotten married. They both were badly beaten multiple times, along with public shaming and psychological abuse.

Mrs. M: “Everyone said that my son has flown away with the girl. Both of them were badly beaten, they were punched in the stomach, I think some 12 stitches were needed because they had hit my son with iron rods. Imagine such brutality only because of caste differences.”

Mrs. V went on to explain how her young daughter was allegedly burned in her marital home. The girl’s face was pushed onto a burning stove, resulting in second-degree burns. Mrs. V was worried for her three unmarried daughters as, despite giving a huge dowry in the elder girls’ marriages, such harrowing incidents continued to plague her family.

Mrs. V: “Her (the daughter) eyes and whole face were swollen, and her cheeks were burnt and were sagging profusely. Thankfully, her ears were saved but the first time that I saw her I almost fainted.”

##### Lack of Social Support Systems

This sub-theme is concerned with the general social environment, where most of the participants preferred to keep limited interactions with other community members, in addition to a perceived lack of sense of belonging to the community. 

Mrs. R reported how most people are ill mannered and that her family can only vouch for themselves.

Mrs. R: “We can’t change people, best is to keep to ourselves, and this is a sign of our helplessness, where can poor people go. People fight here for basic water and necessities. I am scared that no one should get killed in this process.”

Mrs. S does not mingle with other community members, nor does she allow her daughters to do.

Mrs. S: “I don’t like talking to anyone, I only stay at home. I don’t share my life with others in the community and prefer to be by myself as they will only gossip. There are some good people but mostly I believe people are not good-natured.”

#### 3.3.3. Master Theme 3: Sources of Strength and Resilience

This theme explores the sources of resilience to sustain and persist through the challenges. Their belief in God and religion was found to be an important factor, along with inner resilience as means to keep moving ahead for the survival of their families.

##### Religion as Means of Coping

Three out of the six participants described their immense devotion to God for helping them navigate the challenges of their lives. 

Mrs. M was completely immersed in bhakti and devotion through prayers and fasting to cope with the loss of her son. She even mentioned that only God can help her get through her miseries.

Mrs. M: “I want that god takes me into his abode, maybe my life will be successful then. I only recite his name every day and he is the only one who has given me the strength to deal with such a loss. I did not have anywhere to go but him.”

Mrs. T expressed the same sentiment, iterating that she is not alone in this world and only God can give her the strength to fight each day.

Mrs. T: “This is the rule of life, sometimes sadness and sometimes happiness, God will give you the strength to deal with these challenges.”

##### Family as a Meaning of Life

They expressed positive emotions and a sense of hope for what the future holds for them. This was partly true because of their families, and most importantly, the children, who continue to be their reason for survival. They all believed that only quality education could bring some respite from their ongoing struggles and systematically uplift them from poverty.

Mrs. K: “I have seen my mother fight the same way and I have seen the struggles up close. This has given me the strength to fight back and help my children.”

## 4. Discussion

An investigation was conducted to explore the association between various SES indicators and psychological distress in LIEM communities. The findings are presented in terms of risk and protective factors.

### 4.1. Risk Factors

This study aimed to explore psychological distress among LIEM populations of the NCR, India, using a sequential mixed-methods design. The results of the multiple regression analysis show that gender was the most important predictor of psychological distress, with women experiencing more psychological distress than men. As per the cut-off given by Kessler et al. (2003), the women in the present study experienced moderate distress (M = 26.30, SD = 9.15) as compared to the men experiencing mild distress (M = 21.04, SD = 8.35). This resonated with the findings of a study by Coffey et al. (2021) in three states of India (Maharashtra, Bihar, and Jharkhand), in which women were found to have experienced more distress than men [42]. Moreover, gender has also been found to be the second-highest factor influencing mental health for women in slums and low-income spaces [43]. These findings can be attributed to the rigid gender roles and cultural norms that operate for women in many marginalized communities, including India [44]. India is also riddled with gender inequalities that act as barriers for young girls and women to experiencing the same privileges as men concerning health, education, employment, and general dignity [45]. Therefore, they are at a higher risk of developing mental health illnesses [46], especially in low-income and economically marginalized communities.

The results reveal that the age, education, and marital status of the respondents explained psychological distress significantly. Older participants were found to be more distressed. In a study conducted by Joshi et al. (2003), as many as 66% of the older people in the sample were found to be physically and psychologically distressed [47]. Upon exploration of the psychosocial variables influencing mental distress, these findings were attributed to an increase in family responsibilities and a lack of healthcare facilities. Additionally, it has been found that mental health distress is likely to increase with age, with a low-socioeconomic background acting as a major mediating factor [48]. Taking gender into account, in the Indian context, older women have been found to be more distressed than men in the rural areas of Northern India [49]. There is also evidence that age and educational status have significant effects on psychological impairments for older women in the slums of Kolkata, India [50]. In the present study as well, lower education levels of the head of the family predicted psychological distress. There is evidence that higher education and having a job positively correlate with mental health in US and South African samples [51]. Moreover, limited educational qualification has been associated with general high distress and low well-being [52], as well as dementia risk [53]. Within Indian samples, it has been reported that mental health outcomes and vulnerability to developing common mental disorders like depression and anxiety are associated with low education [48]. 

The results also reveal that married respondents experienced less distress than single respondents. A Norway-based sample reported significantly better mental health scores for married people than unmarried people [54]. In the Indian context, recent studies on health workers during COVID-19 revealed that older women and unmarried people reported high mental health stress [55] and had a high risk for anxiety and depression [56]. Similarly, widows who are older adults and lack socioeconomic resources have been found to be at increased risk of developing depression and deteriorating health conditions [48]. Therefore, a multifaceted approach is needed to understand the better mental health outcomes of married individuals [57]. Factors like socioeconomic conditions, demographic factors such as age and gender, and psychological resources like social support might moderate the relationship between marital status and psychological distress. 

The qualitative data analysis was carried out to explore the lived experiences of the participants with regard to psychological distress. All the participants belonged to economically marginalized families with income levels below USD 2.15 per day, as per the International Poverty line for the year 2017 cut-off values [58]. There is a strong relationship between poverty and common mental disorders in low- and middle-income countries [59] There is evidence that being exposed to chronic poverty-related stressors leads to physical and mental health problems [60] like experiencing symptoms of depression, anxiety, suicidality, sleep problems, and somatization [61]. Subbaraman et al. (2014) found that mental health disorders like depression and anxiety were related to poverty-related factors in the slums of Mumbai, India [62]. It was also observed that with the sudden flash of a traumatic incident, four out of six women in the study showed frequent crying spells and unusual sadness, along with bodily complaints. This is supported by the community mental health statistics that point toward lower mental health well-being for women [63,64,65].

In the present study, close to two thirds of the participants (71% to be precise) belonged to the schedule caste community, and this is a reflection of the uneven distribution of wealth in India [66]. The mental health outcomes of lower caste communities in themselves bring forward staggering results that warrant a community-sensitive approach, taking into account the years of collective trauma [67]. For women, this represents a gendered vulnerability to violence and lower mental health well-being, owing to class and caste-based inequalities [32,68] For instance, Prost et al. (2012) found that women belonging to schedule tribes or schedule caste backgrounds reported higher psychological stress than higher-caste women [69]. Intersectionality highlights how various societal factors, such as gender, age, class, and caste, intersect and create a complex web of health inequalities. These inequalities are not isolated but rather reinforce one another, leading to a more entrenched and pervasive system of inequality, thus, in turn, having a multifaceted impact on mental health.

The daily stressors in the community revolve around the plenitude of violent acts leading to personal and collective trauma. The participants narrated incidents of domestic violence, caste- and gender-based violence, and general community aggression. Women in low-income communities also experience high rates of structural and gender-based violence (GBV), besides other sources of stressors. Specifically, Indian women from lower castes, with limited education, are vulnerable to GBV, owing to a lack of financial stability. [70,71]. Another study by Datta and Satija (2020) throws light on gender and caste violence in slums, which they found to be normalized by layers of traditional and newer forms of discrimination like dowry, son preference, and overt forms of physical and sexual violence [72]. Research has repeatedly shown a significant connection between alcoholism and domestic violence [73,74,75] as well as the negative impact that violence can have on mental health [76,77,78]. By recognizing these interconnected issues, we can prevent further harm and promote overall well-being. While social support has been considered a protective factor, the social environment of the participants in the present study did not provide a sense of support, comfort, or belongingness. These findings are similar to Mumbai-based slums that reported around 33% of women do not experience any social support [79]. Moreover, low social and family engagement was found to be a predictor of depression for older adults in rural Allahabad [49]. 

### 4.2. Protective Factors

#### 4.2.1. Religiosity

Despite life’s daily ordeals, all the participants displayed resilience in dealing with the above-mentioned stressors. Religiosity emerged as a means to cope with adversities for many participants. Research has indicated that religious beliefs and practices are associated with fewer anxiety and depression symptoms [80] and overall greater psychological health [81,82]. 

#### 4.2.2. Children and Family as the Meaning of Life

The well-being of their family and the educational pursuits of their children propel them to move forward in life and hope for a better future. Not only does family coping facilitate the management of stressors like job loss, financial crisis, and health issues [83], but it also creates opportunities to break the cycle of intergenerational poverty. The children act as a symbol of meaning-making, a ray of hope for overcoming their existing financial hardships, and most importantly, they help overcome economic constraints. These findings reveal the relevance of family-based psychosocial interventions aimed at fostering resilience in low-income settings.

#### 4.2.3. Limitations

The sample adequacy was established before the data collection; this study was limited to the NCR of India. Therefore, future studies are encouraged to expand across the social–regional diversity of India. Secondly, the in-depth interviews were centered on the lived experiences of the women participants who reported the highest psychological distress; thus, a deeper gender perspective may not have been tapped. Thirdly, this study’s findings on the relationship between caste, socioeconomic status, and mental health issues were limited due to the skewed data as 71% of the participants belonged to scheduled caste communities, which prevented a comprehensive understanding of the systemic caste and class stratification. Further investigation is required to establish the linkages between caste and class stratification. 

## 5. Conclusions

The results of this quantitative investigation reveal that the gender, age, and educational status of the head of the family significantly predicted psychological distress. The qualitative inquiry furthered our understanding of the nature of distress, the context, and the associated symptomology, in addition to the varying coping mechanisms. Moreover, it did so under the lens of the triple vulnerabilities of low income, illiteracy, and gender. This highlighted the intersectionality of the myriad factors influencing SES and psychological distress.

The findings of this research have significant implications for mental health intervention and policies, which can be summarized as follows: 

By establishing appropriate measures of socioeconomic status and social class and understanding the connections between socioeconomic status, caste, and mental health, policymakers can target interventions for those who need them the most. 

This research highlights the importance of mental health interventions that are tailored to the specific experiences of different individuals. There is a possibility of exploring different perspectives and intersections of identity. For example, in the present study, the qualitative interview was carried out with six women participants based on their cut-off scores on the Psychological Distress Scale, but designing interventions that meet the needs of all genders requires understanding the experiences of all. 

This research also sheds light on the impact of caste-related violence and discrimination on mental health. By taking into account the complex relationship between caste, socioeconomic status, class, and mental health, interventions can be designed that are more effective and equitable. 

Finally, given the mental hygiene movement [84] and the need for health interventions to expand the applicability of intersectionality principles [85], notably, the studies at the juncture of development economics and public health take on considerable responsibility in addressing inequality and related mental health distress.

## Figures and Tables

**Table 1:** Showing the sample description.

Variable	Level	Frequency	Percentage
Age	18–76 Mean—32.8, SD—12.43	-	-
Gender	Male	93	48.9
	Female	97	51.1
Religion	Hindu	175	92.1
	Muslim	14	7.4
	Other	1	0.5
Caste ^a^	Scheduled Caste	117	61.6
	Lower Caste	23	12.1
	Artisan Caste	8	4.2
	Agriculture Caste	11	5.8
	General Caste	22	11.6
Marital Status	Married	150	78.9
	Unmarried	40	21.1
Education of the Head of the Family ^b^	Illiterate	38	20
	Primary School	38	20
	Middle School	74	38.9
	High School Certificate	29	15.3
	Intermediate/Diploma	4	2.1
	Graduate	7	3.7
Occupation of The Head of The Family ^b^	Unemployed	22	11.6
	Low-skilled jobs	111	58.4
	Medium-skilled jobs	44	23.1
	High-skilled jobs	13	6.9
Income of the Head of the Family (in rupees) ^b^	<6174	24	12.6
	6175–18,496	154	81.1
	18,496–30,830	8	4.2
	30,830–46,128	3	1.6
	46,129–61,662	1	0.5

^a^ Wani [37], ^b^ Saleem and Jan [36].

**Table 2:** Demographic details of the interview participants.

Name	Age	Religion	Caste ^a^	Education ^b^	Marital Status	Employment ^b^	Income Level ^a^ (INR)
Mrs. M	55	Hindu	SC/ST	Illiterate	Married	Unemployed	6175–18,496
Mrs. K	45	Hindu	SC/ST	Illiterate	Married	Unemployed	6175–18,496
Mrs. S	34	Hindu	SC/ST	Illiterate	Married	Unemployed	6175–18,496
Mrs. R	45	Hindu	SC/ST	Illiterate	Widow	Unemployed	6175–18,496
Mrs. T	37	Hindu	SC/ST	Illiterate	Married	Unemployed	<6174
Mrs. V	45	Hindu	SC/ST	Illiterate	Married	Unemployed	6175–18,496

^a^ Wani [37], ^b^ Saleem and Jan [36]. ECB Exchange rates: 11 December 2023, INR 6175–18,496 = EUR 68.85–206.24; USD 74.07–221.86.

**Table 3:** Showing the correlation coefficient values for all the study variables.

Variable	Mean (SD)	Skewness	Kurtosis	1	2	3	4	5	6	7
1. Age	32.85 (12.43)	0.66	0.06	1	−0.15 *	−0.43 **	−0.30 **	−0.03	0.04	0.20 **
2. Gender	1.51 (0.50)	−0.04	−2.0	-	1	−0.06	0.50	−0.09	−0.13	0.29 **
3. Marital Status	1.21 (0.41)	1.43	0.05	-	-	1	0.22 **	0.10	0.07	0.01
Head of the Family										
4. Education	2.70 (1.22)	0.48	0.27	-	-	-	1	−0.04	−0.04	−0.16 *
5. Occupation	2.99 (2.03)	1.52	1.52	-	-	-	-	1	0.23 **	−0.08
6. Income	1.97 (0.56)	2.33	15.79	-	-	-	-	-	1	−0.09
7. Psychological Distress	23.73 (9.13)	0.53	−0.62	-	-	-	-	-	-	1

Note: Correlation is significant at * 0.05 and ** 0.01 level.

**Table 4:** Multiple regression of variables predicting psychological distress.

Variable	B	Std. Error	β	t	*p*
Age	0.21	0.06	0.29	3.79	*** <0.001
Gender	6.2	1.2	0.34	4.95	*** <0.001
Marital Status	4.5	1.7	0.20	2.67	** 0.008
Head of the Family					
Education	−1.07	0.53	−0.14	−2.03	* 0.04
Occupation	−0.23	0.31	−0.05	−0.76	0.45
Income	−1.11	1.12	−0.07	−0.99	0.32

R = 0.44, R^2^ = 0.19, F (6183) = 7.23, *p* < 0.01. Significant at * 0.04, ** 0.008, and *** <0.001.

**Table 5:** Overview of the master themes and subordinate themes.

Master Themes and Subordinate Themes
1. Manifestation of Psychological Distress Physiological disturbances and bodily concerns Emotional and psychological disturbances
2. Contextual challenges
Experiences of living in abject poverty A spectrum of violence and traumatic incidents Lack of social support systems
3. Sources of strength and resilience
Religion as means of coping Family as a ray of hope

## Data Availability

The original contributions presented in the study are included in the article, further inquiries can be directed to the corresponding author.
